# Safety and tolerability evaluation after repeated intravitreal injections of a humanized anti-VEGF-A monoclonal antibody (PRO-169) versus ranibizumab in New Zealand white rabbits

**DOI:** 10.1186/s40942-020-00235-y

**Published:** 2020-07-28

**Authors:** Leopoldo Baiza-Durán, Alejandra Sánchez-Ríos, Joel González-Barón, Oscar Olvera-Montaño, Elba Correa-Gallegos, Andrea Navarro-Sánchez, Patricia Muñoz-Villegas

**Affiliations:** 1 Medical Affairs; Laboratorios Sophia, SA de CV, Paseo del Norte 5255, Guadalajara Technology Park, Zapopan, Jalisco Mexico; 2 Clínica Lacandones, Guadalajara, Jalisco Mexico

**Keywords:** Anti-vascular endothelial growth factor, Electroretinogram, Naka-Rushton function, Retina neovascularization

## Abstract

**Background:**

To evaluate the retinal toxicity after repeated intravitreal injections of a humanized anti-VEGF-A monoclonal antibody (PRO-169) versus ranibizumab in New Zealand white (NZW) rabbit eyes.

**Methods:**

NZW rabbits were injected intravitreally with PRO-169 (n = 12), 1.25 mg/0.05 ml or ranibizumab (n = 12), 0.5 mg/0.05 ml into the right eye (OD), whereas the left eye (OS) of each rabbit was used as control. Three consecutive injections were administered at 30-days intervals. An electroretinogram (ERG) was recorded 30 days after each injection. Clinical examination was conducted before and after injections, including intraocular pressure determination and eye fundus exploration. Eyes were enucleated and retina, cornea, conjunctiva, ciliary body and optic nerve were prepared for histopathology assessment.

**Results:**

ERG of the experimental and control eyes in PRO-169 and ranibizumab groups were similar in amplitude and pattern throughout the follow-up period. Clinical examination found no alterations of intraocular pressure (IOP). No retinal damage was observed in both, the experimental and control eyes, of all the rabbits. The histopathologic studies showed similar results in both groups, showing no signs of structural damage.

**Conclusions:**

Our study did not find evidence of retinal toxicity from a repeated intravitreal injection of PRO-169 or ranibizumab (Lucentis^®^) in NZW rabbits. These findings support intravitreal PRO-169 as a safe candidate to develop as a future alternative for the treatment of retinal neovascularization diseases.

## Background

Vascular endothelial growth factor (VEGF) promotes angiogenesis and neovascularization, regulating vascular differentiation and permeability [1]. Its presence is necessary in order to maintain the normal functions of the eye, yet it can be harmful when it is overproduced, as it happens in diseases whose pathophysiology is based on neovascularization of the retina. Diabetic retinopathy (DR), age-related macular degeneration (ARMD), retinopathy of prematurity (ROP) and central retinal vein occlusion (CRVO) are some of such diseases; and their prevalence is a real public health threat [2–4]. DR alone affects up to 80% of patients with chronic Diabetes Mellitus, while diabetic macular edema is diagnosed in 16% of them [5, 6].

To inhibit VEGF, many anti-VEGF agents have been developed, and their use has increased dramatically over the last decade. Bevacizumab is a humanized monoclonal antibody (mAb) approved by the Food and Drug Administration (FDA) for metastatic colorectal cancer, which has also been used extensively as an off-label intravitreal treatment for many neovascular related retinal conditions. PRO-169 is a recombinant, humanized anti-VEGF-A mAb with a molecular mass of 149 kDa, structurally similar and with a target specificity like bevacizumab (Avastin, Genentech, South San Francisco, CA) [7, 8]. The peptide map of PRO-169 is consistent with bevacizumab’s which confirms its identity, and the structure is typical of an IgG_1_ antibody predominantly comprised of parallel β-sheets. The vitreous pharmacokinetics of a single intravitreal injection of PRO-169 have been evaluated by surface plasmon resonance (SPR) in a preclinical study in NZW rabbits. PRO-169 has a similar pharmacokinetic profile to commercially available bevacizumab, see Table 1. Ranibizumab (Lucentis, Genentech) is a fab fragment from bevacizumab’s mAb. The major differences between bevacizumab and ranibizumab are their molecular weights (149 vs 48 kDa, respectively) and the number of VEGF binding sites (2 vs 1) on each. Both drugs have demonstrated equivalent clinical effects as ARMD treatment throughout the first year of follow-up [9, 10]. Bevacizumab’s effectiveness and safety profile has been confirmed through many preclinical studies, including in vivo studies in numerous species. Many of these have shown that the repeated intravitreal injections of bevacizumab or ranibizumab have no long-term deleterious effects on the electrophysiological and morphologic integrity of the retina [1, 11–17]. Another preclinical in vivo study reported that after Choroidal Neovascularization (CNV) induction through retinal photocoagulation, PRO-169 administration (1.25 mg per eye) can inhibit the retinal thickness and fluorescein leakage area without toxic effect or adverse events in a rhesus monkey model [4]. Assessing retinal toxicity of PRO-169, through controlled research in an animal model using ERG and clinical tests after repeated intravitreal (Ivt) injections was needed. Long-term VEGF suppression may produce a toxic effect on the retina. Repeated monthly Ivt injections of bevacizumab or ranibizumab over a three-month period have no cumulative toxic effect on the retina in rabbits as judged by the electroretinogram (ERG) [1, 13].

**Table 1:** Characterization of PRO-169

Test	Method	PRO-169
Quality	Appearance	Slightly yellow, opalescent liquid
	pH	6.2
Charge of heterogeneity	CEX-HPLC	55.1% main peak, 34.3% acidic variants, and 10.7 basic variants
Structure	Intact mass LC/MS, G0F/G0F	149201 Da
	Deglycosylated partially reduced LC/MS	Light chain mass = 23450.8 Da, deglycosylated heavy chain mass = 49717.5 Da
Oligosaccharide profile	N-Glycan profiling	79.2% G0, 16.1% G1, and 0.9% G2
Other	SPR	K_D_ = 6.54 × 10^−6^
Vitreous Pharmacokinetics	SPR	C_Max_ ± SD = 593.7 ± 42.6 µg/ml
		T_Max_ ± SD = 0.53 ± 0.8 days
		T_1/2_ ± SD = 4.99 ± 0.9 days

CEX-HPLC: cation exchange chromatography-high performance liquid chromatography; C_Max_: peak maximum concentration; K_D_: binding constant; LC/MS: liquid chromatography/mass spectrometry; SD: standard deviation; SPR: surface plasmon resonance; T_1/2_: half-life time; T_Max_: time to maximal concentration

The purpose of this in vivo preclinical study was to assess the retinal toxicity after repeated intravitreal injections of a humanized anti-VEGF-A monoclonal antibody (PRO-169) versus ranibizumab (positive control) in New Zealand White (NZW) rabbit eyes.

## Methods

### Animals

**Fig. 1:**
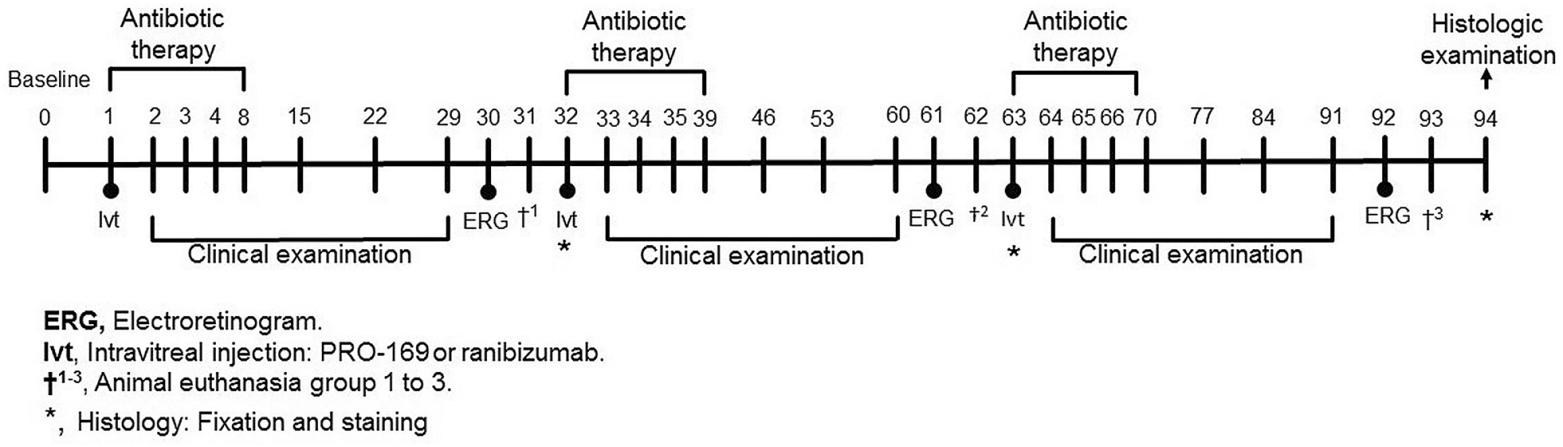
Study design. In total, 24 NZW rabbits were included in this study

A total of 24 experimental animals were included in this study. Inclusion criteria were: healthy male NZW rabbits aged 2 to 3 months and weighing between 2 and 3 kg, with no history of participation in any previous study. All subjects were submitted to at least 7 days of quarantine, in which general health was assessed, and, weight, food and water uptake were registered. An ophthalmic eligibility screening with slit lamp examination and fluorescein staining was performed to ensure there were no exclusion criteria present, such as: secretion, conjunctival hyperemia, corneal or conjunctival lacerations, de-epithelization or scarring, corneal degeneration or neovascularization, cataract, reduction in aqueous humor transparency, or any pathological findings in the indirect fundoscopy performed with a 78 D lens, including retinal detachment, tears or neovascularization. Finally, the elimination criteria included any serious adverse event that required the administration of a complementary ophthalmic or systemic treatment, including any situation that entailed any compromise to the animal’s well-being. All animal studies were conducted according to the ARVO Statement for the Use of Animals in Ophthalmic and Vision Research; they were approved by Institutional Animal Care and Use Committee of Laboratorios Sophia, SA de CV (CICUALLS). The NZW rabbits were housed under a 12/12-h light/dark cycle with free access to food and water. Slit-lamp and indirect funduscopic examinations were performed on all eyes before the study began, and on days 2, 3, 4, 8, 15, 22, 29, 33, 34, 35, 39, 46, 53, 60, 64, 65, 66, 70, 77, 84 and 91; and Ivt injections took place on days 1, 32 and 63 (n = 4 NZW rabbits per group), see Fig. 1. One rabbit (PRO-169 group) died during the follow-up period (after the 3^rd^ Ivt injection, day 92), and therefore only data from 23 rabbits was used for the histologic examination.

### Anesthesia procedure

Before Ivt injection and electrophysiological testing, the animals were anesthetized with an intramuscular injection of 10 mg/kg body weight of xylazine (PROCIN^®^ Pisa, Hidalgo, Mexico), and 30 mg/kg of ketamine hydrochloride (ANESKET^®^ VET, Pisa, Hidalgo, Mexico). Topical anesthesia (tetracaine hydrochloride 0.5%, Ponti^®^ Ofteno, Laboratorios Sophia, Zapopan, Jalisco, Mexico) was administered to prevent any potential discomfort.

### Intravitreal injection

Before Ivt injection, animals were subjected to an asepsis and antisepsis protocol for eyelid and eyelash with an iodate solution (50%) (Isodine^®^, Boehringer Ingelheim, Germany) plus a drop of iodate solution (5%) placed in the conjunctival sac. Test article, PRO-169 is a mAb Anti-VEGF (Laboratorios Sophia, SA de CV, Zapopan, Jalisco, Mexico by KBI Biopharma). Positive control, ranibizumab (Lucentis^®^, Novartis Pharmaceuticals, Genentech Inc, San Francisco, CA, USA) is a commercially available monoclonal antibody; Ivt injections were performed under sterile conditions. A 30-gauge needle attached to a syringe containing test or control article (PRO-169 1.25 mg/0.05 ml or ranibizumab 0.5 mg/0.05 ml into the right eye) was used for Ivt injection through an area 1.5–2 mm posterior to the limbus, inside the vitreous cavity in the superonasal quadrant. The eye was held by 0.12 forceps during the procedure. Injection was done slowly with the bevel of the needle pointing away from the retina to avoid any undesired mechanical damage. At the end of the procedure, a broad-spectrum antibiotic eye drop was applied four times per day for seven days (ciprofloxacin 0.3%, Sophixin^®^ Ofteno, Laboratorios Sophia, SA de CV, Zapopan, Jalisco, Mexico).

### Electroretinogram (ERG)

Electroretinography using the UTAS-3000 system (LKC Technologies, Gaithersburg, MD) was performed 30 days after each injection. The ERG responses were recorded simultaneously from the experimental (OD) and control (OS) eyes. The rabbits were dark adapted for at least 30 min after pupillary dilation (~ 8 mm). ERG-jet electrodes (Universe SA, Switzerland) were placed on both corneas after applying methylcellulose 2% (Meticel^®^ Ofteno, Laboratorios Sophia, SA de CV, Zapopan, Mexico), the negative electrode was placed on a shaved forehead’s section, and a ground electrode was clipped on the subject’s ear (Grass^®^ electrodes, USA). Light signals were obtained from a Ganzfeld optoelectronic stimulator (Universial, Metrovision) [10]. A homologated procedure was performed with different flash intensities (−35, −30, −25, −20, −15, −10, −3, 0 and 3 dB), ERGs were recorded with a standard white flash without attenuation and a scotopic background. Three to ten responses elicited by identical flashes applied at 4–30-s intervals were averaged in the dark-adapted state. Each ERG was performed by a doctor who was blinded to the treatment groups to minimize the observer bias. ERG analysis was based on amplitude measurements of the b-waves. Dark adapted rod and mixed ERG responses were obtained in order to assess the linear scotopic rod function, however, some interaction from the cone function was expected since it is impossible to avoid it completely. Amplitudes were measured from baseline to cornea-positive peak. For each rabbit, the amplitudes of the experimental and control eyes were plotted as a function of log light energy. The response-stimulus energy relationship was fitted to a Naka Rushton-type hyperbolic function [1, 11, 12, 18].

1 $$ V/V_{\text{Max}} = I\left( {I + \sigma } \right) $$

where V is the amplitude of the b-wave elicited by a stimulus of energy I (cd s/m^2^), V_Max_ is the maximum response asymptotic amplitude of the b-wave (µV), and σ is the semisaturation constant. For each stimulus intensity response, the mean V/V_Max_ was calculated for the injected eyes and compared with the V/V_Max_ for the control eyes (not injected). The amplitude ratio (experimental/control) served as an index of PRO-169 and ranibizumab effect on photopic retinal function. To assess retinal response, the data obtained at each ERG recording session was derived to the entire response-stimulus energy relationship and the V_Max_ ratio (experimental/control eye) and the semi saturation constant difference (experimental–control) of the dark-adapted b-waves were calculated [19]. Previous studies have reported that with this approach, technical factors such as depth of anesthesia and duration of adaptation did not affect the evaluation of retinal function [12, 13, 20].

### Clinical observation

Evaluations were performed 7 times after each Ivt injection. The posterior segment was evaluated by slit lamp (Luxvision^®^, Class I Type B, Doral FL, USA), and an exploration 78 D lens (Ocular Instruments, Belleveu, WA, USA). The structures involved during this evaluation were; vitreous, retina, macula, fovea, choroid, optic nerve, and blood vessels. Without changing the slit-lamp settings, the anterior chamber was examined for the presence of cells (0.5+ at 4.0+). The intraocular pressure (IOP) was measured with Goldmann tonometer (Luxvision^®^, YZ30, Doral FL, USA).

### Histopathologic evaluation

The animals were euthanatized after the ERG (on the 31th [n = 8], 62nd [n = 8] and 93th day [n = 7]) by intravenous injection of 100 mg/Kg body weight of pentobarbital (Doléthal^®^, Vétoquinol, SA, Distrito Federal, Mexico). An ophthalmologist performed a bilateral enucleation of all twenty-three NZW rabbits [21]. The enucleated eye was fixed for 24–48 h in a solution of 4% paraformaldehyde. Paraffin sections of 4 µm were prepared, stained with hematoxylin, eosin and AAPas for examination with light microscope. The goblet cells were counted using high power field (×40).

### Statistical analyses

Statistical analyses were carried out using SPSS 19.0 software for Windows (SPSS Inc., Chicago, IL, USA). Microsoft^®^ Office Excel 2016 was used for data processing. MATLAB^®^ 2019a software for Mac (MathWorks Inc^®^, Natick, Massachusetts, USA) was used for data analyses on ERGs. Statistical significance was determined by Mann–Whitney test for continuous data, and Chi square test or Fisher’s exact test for categorical data. All statistical analyses performed in this study were with *p* values ≤ 0.05 considered statistically significant.

## Results

### Electroretinogram

**Fig. 2:**
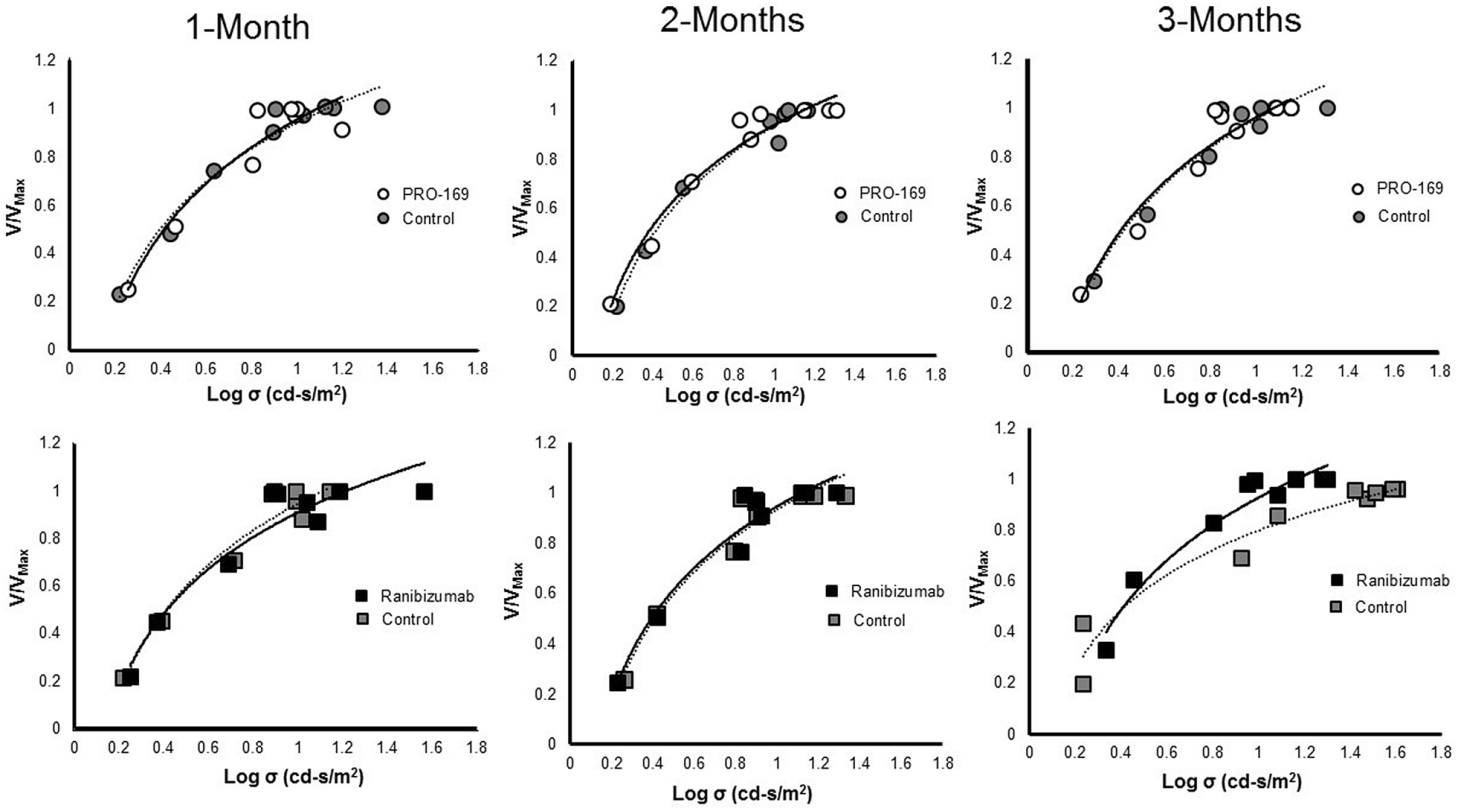
Response-stimulus strength for the dark-adapted ERG b-wave for each ERG recording session (1, 2 and 3-months), from experimental (OD) and the control (OS) eyes. The relationships were fitted to hyperbolic function (Eq. 1) to derive the maximal amplitude response (V/V_Max_) and the semi saturation constant (σ). For PRO-169 and ranibizumab at each recording session, the response-stimulus energy relationship of the control and experimental eyes are similar

**Fig. 3:**
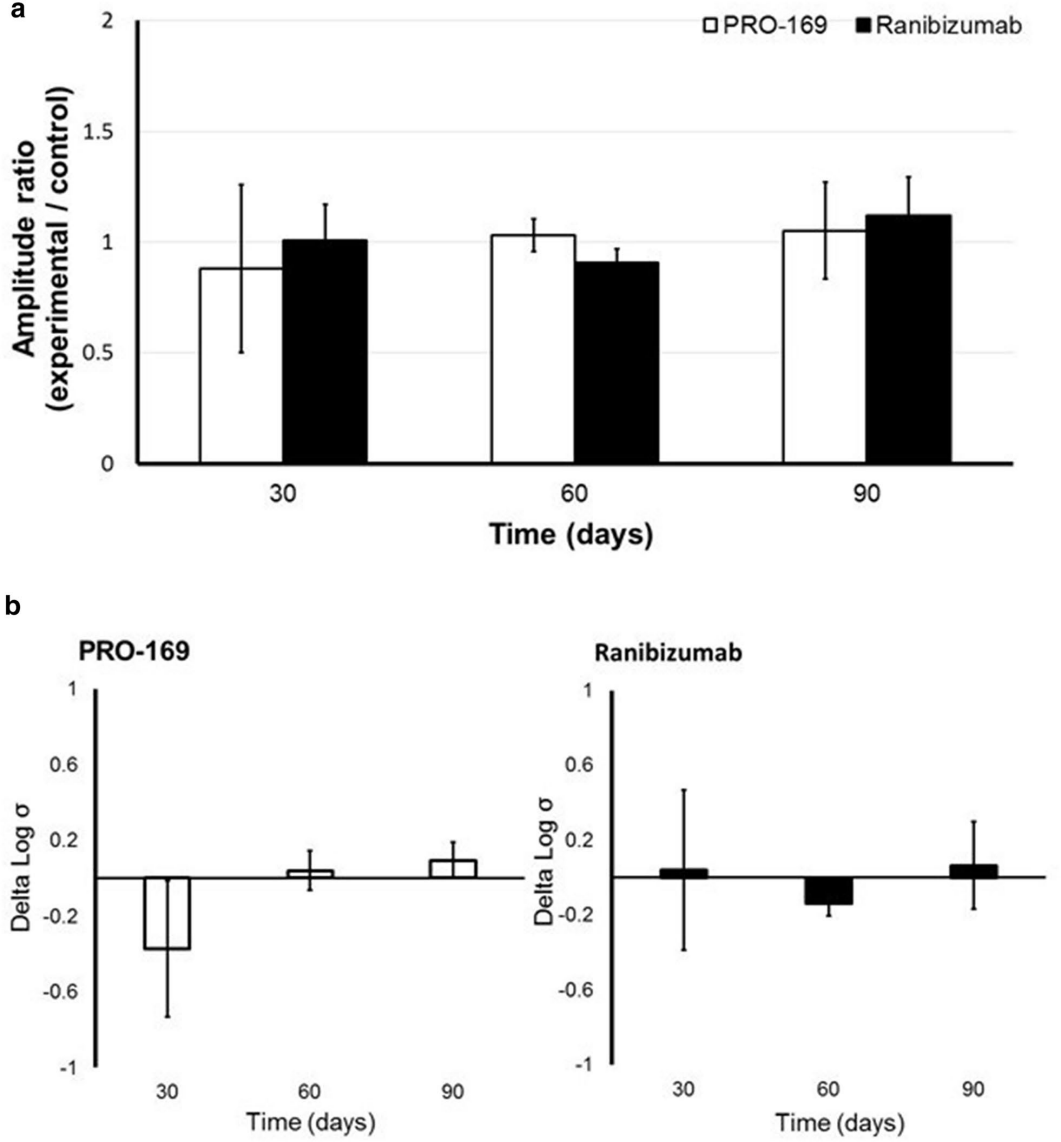
ERG analysis of NZW rabbits as a function of time after intravitreal injections. **a** The effects of the intravitreal PRO-169 (white bars) and ranibizumab (dark bars) on the dark-adapted ERG responses after 30 days of each injection. The dark-adapted retinal response is represented by the mean ± SEM V_Max_ ratio of the ERG (b-wave). **b** Time dependent effects of repeated injections of PRO-169 and ranibizumab on retinal function of rabbits. The difference of the log semi saturation constant (experimental-control) is represented by the mean ± SEM. V_Max_ ratios are around 1 and log σ differences are around 0, indicating no damage to the road system

Dark-adapted ERG responses from the experimental (OD) and control (OS) eyes were similar in pattern and amplitude through the follow-up period. During the recording sessions, different flash intensities were used to derive the response-log stimulus strength relationship. Similar results were noted for animals treated with repeated injections of PRO-169 or ranibizumab, as represented by ERG response-stimulus energy relationship (V/V_Max_/Log σ), as shown in Fig. 2. ERG changes were considered significant if the follow-up differences in amplitude (b-waves) were over 20% inferior in the test article (PRO-169) group when compared to the positive control (ranibizumab). Both groups only showed a decrease in amplitude at day 60 (after 2 Ivt injection) of 32.3% for PRO-169 and 12.7% in ranibizumab when compared to day 30 (p = 0.386). This study did not show a significant decrease in amplitude in both groups at day 90 when compared to day 60 (p = 0.386), and at day 90 when compared to the day 30 (p = 0.564). No significant differences in retinal response were found between the PRO-169 and ranibizumab groups at any time point, expressed in V_Max_ (p-values: 0.248, 1.000, 0.248 on day 30, 60 and 90, respectively) and σ values (p-values: 0.486, 0.858 and 0.384 on day 30, 60 and 90). The effects of the PRO-169 and ranibizumab on the dark-adapted retinal responses are represented in Table 2 and showed in Fig. 3a. The mean ± standard deviation (SD) for the V_Max_ ratio of the ERG b-wave for the PRO-169 group were 0.879 ± 0.755, 1.031 ± 0.148 and 1.050 ± 0.378 at 30, 60 and 90 days respectively; meanwhile for the ranibizumab group they were 1.007 ± 0.328, 0.907 ± 0.123 and 1.118 ± 0.347 (p-values: 0.248, 0.248 and 1.000, respectively). No electroretinographic deficit was found for the two treatments across the follow-up period, the dark-adapted b-wave V_Max_ ratio of the maximal amplitudes was close to unity and the difference in the log semi saturation constant of the dark-adapted ERG b-wave was small and close to zero in 30, 60 and 90 days after the Ivt injection. See Fig. 3b.

**Table 2:** ERG’s Maximum b-wave amplitude and semi saturation constant from the rabbits treated with PRO-169 and Ranibizumab intravitreal injections

	Experimental eye		Control eye		V_max_ ratio	δ Log σ
	V_max_ (µV)	Log σ (cd s/m^2^)	V_max_ (µV)	Log σ (cd s/m^2^)		
PRO-169						
1 month	128.747	− 2.626	154.359	− 2.253	0.879	− 0.373
2 months	276.490	− 2.313	266.438	− 2.353	1.031	0.040
3 months	286.428	− 2.473	238.032	− 2.756	1.050	0.094
Ranibizumab						
1 month	135.376	− 2.177	136.858	− 2.218	1.007	0.041
2 months	268.382	− 2.468	299.659	− 2.332	0.907	− 0.136
3 months	241.647	− 2.696	229.543	− 2.760	1.118	0.064

Medium values. No significant differences between experimental (OD) and control eyes (OS) and between groups (injected eyes), in all comparisons, p > 0.05 (Mann–Whitney U test), n = 4 NWZ rabbit’s eyes per group

### Clinical observation

100% of the eyes examined in both treatments presented absence of any pathological condition, active or inactive in retina, macula, fovea, choroid, optic nerve and, blood vessels. Evidence of eye inflammation was seen in the anterior segment of ranibizumab injected rabbit eyes after the 3rd Ivt (64 at 77 day), no significant differences were observed (p = 1.000). Ophthalmic examination revealed an appearance of the cells in the anterior chamber (mild to moderate) in 2 of 8 eyes which received PRO-169 and 2 of 8 eyes which received ranibizumab. There was no significant difference in the cellularity described in the anterior chamber between both groups. Additionally, one eye in the PRO-169 group (46 at 60 day) and one eye in ranibizumab group (D29) presented posterior vitreous detachment.

Ocular tonometry showed no significant differences in the IOP between PRO-169 and ranibizumab groups after 30 (8.75 ± 0.4 vs 8.92 ± 0.5 mmHg; p = 0.422), 60 (8.63 ± 0.5 vs 8.75 ± 0.5 mmHg; p = 0.602) or 90 days (8.50 ± 0.6 vs 8.75 ± 0.5 mmHg; p = 0.495).

Finally, no subjects presented cataract formation after intravitreal application of either product. The incidence of adverse events was similar between groups.

### Histopathologic evaluation

**Fig. 4:**
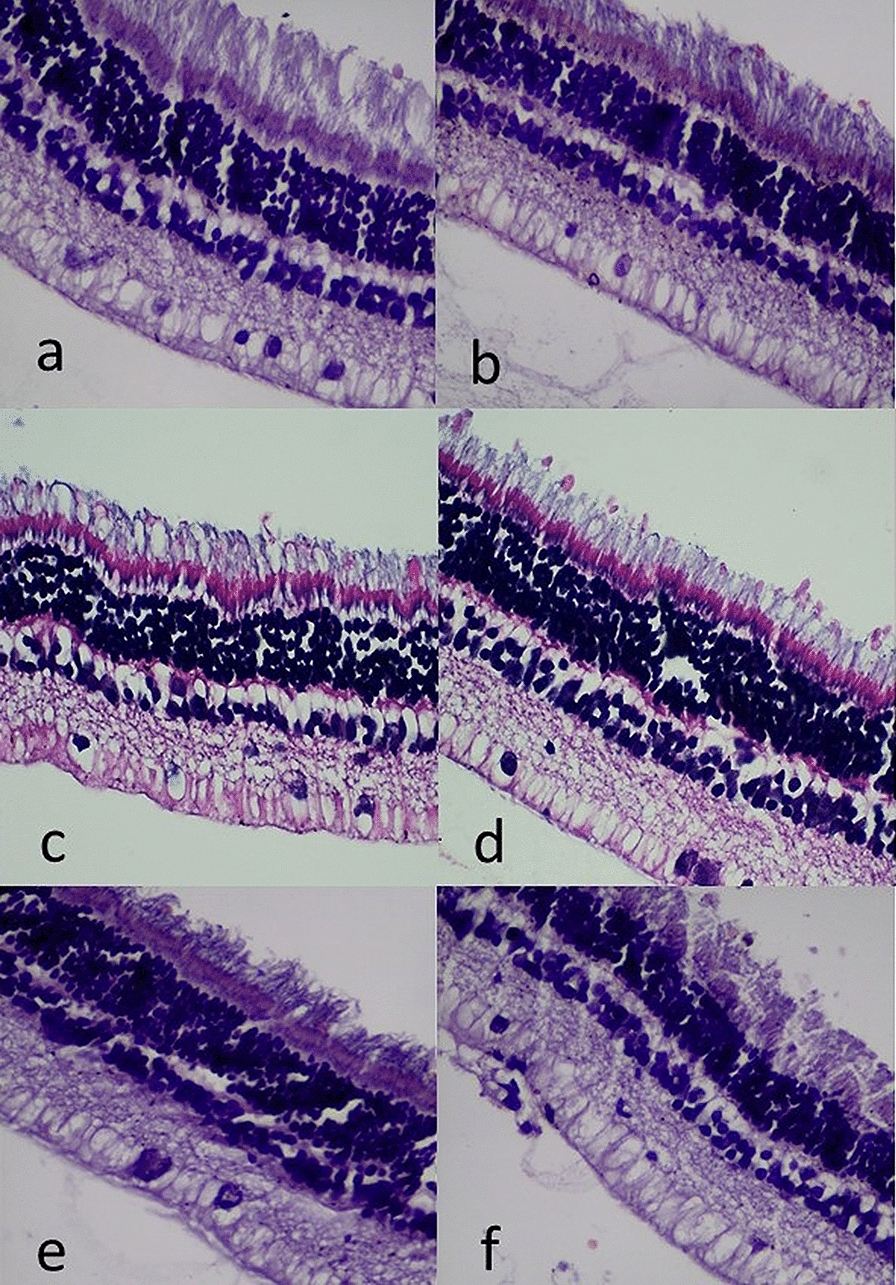
Histologic examination of the central retina for PRO-169 (**a**) vs ranibizumab (**b**) after the 1st Ivt injection (D32), for PRO-169 (**c**) vs ranibizumab (**d**) after the 2nd Ivt injection (D63), and for PRO-169 (**e**) vs ranibizumab (**f**) after the 3rd Ivt injection (D94). No retinal toxicity was found in any eyes (x40)

Light microscopy was performed in all eyes (experimental and control). No retinal toxicity was found in any eyes. The histology of both groups of treated eyes (PRO-169 and ranibizumab) after Ivt injections did not show anatomic signs of toxicity or structural damage, see Fig. 4a–f. In addition, no significant differences were observed in the number of goblet cells (AAPas  %) between treatments, after 30 and 90 days (p = 0.762, p = 0.856). After 60 days, the number of goblet cells on the PRO-169 group was statistically lower when compared to ranibizumab (p = 0.037).

## Discussion

There is a large number of patients who suffer angiogenesis related diseases such as DR, which affects up to 80% of patients with chronic Diabetes Mellitus, and diabetic macular edema. Anti-VEGFs are among the treatment options for such patients [3, 8]. These agents inhibit VEGF, an important angiogenesis and neovascularization regulator. VEGF is found in conditions such as ARMD, DR, ROP, etc. Bevacizumab is a humanized mAb widely injected intravitreally to treat these diseases, even if this administration route and indication is currently considered off-label. Many studies have been executed to confirm Bevacizumab’s safety, including preclinical in vivo studies (NZW rabbits, mice, rats, Dutch rabbits, Cynomolgus monkeys, etc.) [1, 11–16, 20]. The main variable in these is usually the retinal response as studied by ERG and anatomical integrity evaluated through histopathological evaluation. Studies of the ERG pattern of rabbits have proven that the use of interindividual control eyes for testing toxicity is more sensitive, where variability (relative 95% limits of agreement) is 10% as opposed to 33% between individuals, and 20% across sessions [22].

PRO-169, is a mAb structurally like bevacizumab on target specificity, identity and pharmacokinetics, specific for ophthalmic use. On a laser-induced CNV rhesus monkey model, it demonstrated to be able to reduce the retinal thickness and fluorescein leakage area after treatment for 2 and 4 weeks, without toxic effect or adverse events [4]. However, controlled research in an animal model using electrophysiological and clinical tests after repeated intravitreal injections was needed. This research was designed to assess retinal toxicity of PRO-169 versus ranibizumab (positive control), administered as repeated Ivt injections in a NZW rabbit model.

The main outcome ERG variable considered in this study was V_Max_, assessing the integrity of the photoreceptors through the saturation of the b-wave. The V/V_Max_ relationship was also studied to evaluate the integrity of the overall retina’s sensibility. The Naka-Rushton equation describes the empirical amplitude of the adapted wave (b-wave) as a function of the luminance stimulus. This equation was adapted accordingly to fit the ERG’s parameters [1, 11, 13]. Both V_Max_ and σ values, evaluating amplitude and sensitivity respectively, showed no significant differences when comparing both treatments (p˃0.05). The only difference between both groups appeared as a decrease of 32.3% in amplitude at day 60 for PRO-169, and 12.7% for ranibizumab when compared to day 30 (p = 0.386). There was no evidence of a significant decrease in amplitude in both groups at day 90 when compared to day 60 (p = 0.386), and at day 90 when compared to the day 30 (p = 0.564).

The clinical observation included a complete slit lamp evaluation of the anterior segment and IOP, as well as a fundus evaluation during a total of 7 visits after each intravitreal injection. The outcome variable for safety was the presence of cellularity in the anterior chamber as a sign of inflammation. This cellularity was described (mild to moderate) in 2 of 8 eyes after receiving PRO-169 and 2 of 8 eyes in the ranibizumab group. There was no significant difference in the amount of cells described in the anterior chamber between both groups. Due to the nature of the administration method, these findings were attributed to inflammation secondary to the intravitreal injection itself, since this is an invasive procedure and such events have been described before for this route of administration [23, 24]. Additionally, the deceased subject on day 92 belonged to the PRO-169 three-applications group. It did not present any clinical finding that suggested illness prior to its death, the necropsy indicated that was non-related to treatment.

Finally, histopathological evaluation of the enucleated eyes was performed. After fixation and staining with hematoxylin, eosin and AAPas there was no evidence of toxicity or any significant difference in the findings between subjects of both groups. Other authors [12, 16, 25], have evaluated the toxicity in NZW rabbits’ enucleated eyes after exposure to bevacizumab and other anti-VEGF molecules through histologic analysis including hematoxylin and eosin (H&E) staining. Comparably to our study, no significant difference in retinal toxicity were reported between experimental and control groups. And the incidence of adverse events was similar between PRO-169 and ranibizumab groups.

The limitations of this study were the model used, the scarce number of subjects in each group per product and number of intravitreal injections administered. The albino condition of the species used does modify the retinal response in the ERG due to scatter and reflection at the retinal layer [19, 20]. However, the fact that this was a comparative evaluation between PRO-169 and ranibizumab in the same animal model parallels the results and attests to the value of the correlation of the studied parameters. It is also worth mentioning that NZW rabbits have been used amply to assess the safety of this kind of medication intravitreally administered medications by other authors, providing valuable information comparable to that shown on this study. On regards of the small number of subjects used, a resource equation method was used to calculate the sample size with the purpose of avoiding to use a number of animals greater than the strictly necessary to obtain reliable data [26, 27]; especially for the study of pharmaceuticals with an acknowledged broad safety profile such as these.

## Conclusion

In conclusion, all the variables studied in this preclinical study, including ERG responses, clinical evaluation and histopathological findings confirm that the safety profile of the anti-VEGF PRO-169 is comparable to that of ranibizumab, the currently commercially available molecule approved for intravitreal injection as treatment for patients with neovascular conditions affecting their retina.

## Data Availability

The datasets generated and analyzed during the current study are available in Open Science Framework (https://osf.io) at 10.17605/osf.io/n8thw.

## Abbreviations

ARMD: Age-related macular degeneration
CNV: Choroidal neovascularization
DR: Diabetic retinopathy
CRVO: Central retinal vein occlusion
ERG: Electroretinogram
IOP: Intraocular pressure
Ivt: Intravitreal
mAb: Monoclonal antibody
NZW: New Zealand white
OD: Right eye
OS: Left eye
OP: Retinopathy of prematurity
VEGF: Vascular endothelial growth factor
